# CO_2_ Reforming of Methane over Ru Supported Catalysts Under Mild Conditions

**DOI:** 10.3390/molecules30102135

**Published:** 2025-05-12

**Authors:** Alexandros K. Bikogiannakis, Andriana Lymperi, Paraskevas Dimitropoulos, Kyriakos Bourikas, Alexandros Katsaounis, Georgios Kyriakou

**Affiliations:** 1 Department of Chemical Engineering, University of Patras, 26504 Patras, Greece; bikos@chemeng.upatras.gr (A.K.B.); lymperi@chemeng.upatras.gr (A.L.); parisdim@iceht.forth.gr (P.D.); alex.katsaounis@chemeng.upatras.gr (A.K.); 2 Institute of Chemical Engineering Sciences, Foundation for Research and Technology, 26504 Patras, Greece; 3 School of Science and Technology, Hellenic Open University, 26335 Patras, Greece; bourikas@eap.gr

**Keywords:** carbon dioxide, methane, DRM, Ru, XPS, CO_2_ reforming

## Abstract

The CO_2_ (Dry) Reforming of Methane (DRM) is a key process for reducing CO_2_ and CH_4_ emissions while producing syngas with an H_2_/CO ratio of 1, ideal for Fischer–Tropsch synthesis. This study explores DRM and the Reverse Water Gas Shift (RWGS) reaction under mild conditions using Ru-based catalysts supported on CeO_2_, YSZ, TiO_2_, and SiO_2_, with three reactant ratios: (i) stoichiometric, PCO_2_ = 1 kPa, PCH_4_ = 1 kPa, (ii) oxidizing, PCO_2_ = 2 kPa, PCH_4_ = 1 kPa, and (iii) reducing, PCO_2_ = 1 kPa, PCH_4_ = 4 kPa. The results highlight the importance of redox support for catalyst stability, with mobile lattice oxygen aiding carbon gasification. While Ru/CeO_2_ is stable at high temperatures, it rapidly deactivates at low temperatures, emphasizing the need for precise metal particle size control. This work demonstrates the necessity of fine-tuning catalyst properties for more sustainable DRM, offering insights for next-generation CO_2_ utilization catalysts.

## 1. Introduction

The sharp increase of atmospheric greenhouse gas emissions, particularly carbon dioxide (CO_2_) and methane (CH_4_), prompted 195 countries to adopt the Paris Agreement in December 2015 [1]. The agreement aims to limit the increase in the global average temperature to below 2 °C relative to the pre-industrial levels. Despite these efforts, CO_2_ and CH_4_ concentrations reached new record highs in 2023, at 420 ppm and 1934 ppm respectively [2]. Given that both gases are abundant and accessible carbon sources, whether captured from the atmosphere or industrial emissions, their utilization for the production of valuable chemicals and fuels offers a promising strategy for reducing carbon footprints [3].

CO_2_ emissions primarily originate from electricity and heat production, as well as fossil fuel-powered transportation. In contrast, CH_4_ emissions mainly stem from agriculture, particularly rice cultivation along with waste and biomass treatment. Interestingly, anaerobic biological waste treatment can lead to the generation of biogas, a mixture composed mainly of CH_4_ (50–75%) and CO_2_ (25–50%). Biogas is essentially a renewable power source due to its high concentration of methane and is used in internal combustion engines for heat production [4,5,6]. Alternatively, biogas can also be used directly as feedstock for the production of value-added products via the Dry Reforming of Methane (DRM) reaction (Equation (1)).

DRM processes simultaneously convert CO_2_ and CH_4_ into hydrogen (H_2_) and carbon monoxide (CO), a mixture known as synthetic gas (syngas), with a H_2_/CO ratio equal to 1.

(1) $$CH_{4}+CO_{2}\to 2\;CO+2\;H_{2},\Delta H_{298K}^{0}=248\;kJ\;mol^{-1}$$

Syngas, a key industrial gas mixture, is primarily used in ammonia synthesis through the Haber–Bosch process and as a feedstock for the production of higher hydrocarbons and alcohols though the Fischer–Tropsch reaction [7,8,9,10]. The associated Reverse Water Gas Shift (RWGS) reaction (Equation (2)) is mildly endothermic and thermodynamically favored at lower temperatures. It consumes part of the produced H_2_ through the DRM reaction, leading to additional production of CO and thus altering the H_2_/CO ratio [11,12].

(2) $$CO_{2}+H_{2}\to CO+H_{2}O,\Delta H_{298K}^{0}=41\;kJ\;mol^{-1}$$

The DRM reaction is highly endothermic, requiring high temperatures (T > 700 °C) to achieve desirable reactant conversion and high selectivity [13]. However, such conditions require considerable energy input, leading to increased operational costs, and can also be fatal to the catalyst over a long period of time, either due to metal particle sintering or coke deposition that can significantly reduce the active sites of the catalyst. Carbon formation is mainly attributed to the Boudouard reaction (Equation (3)) favored at temperatures < 700 °C and the CH_4_ decomposition reaction (Equation (4)) favored at temperatures > 550 °C [14,15].

(3) $$2\;CO\to C+CO_{2},\Delta H_{298K}^{0}=-172\;kJ\;mol^{-1}$$

(4) $$CH_{4}\to C+2\;H_{2},\Delta H_{298K}^{0}=75\;kJ\;mol^{-1}$$

It is evident that the commercialization of the DRM reaction is still far from feasible [16]. Advancements in catalytic systems are essential to prevent catalyst deactivation due to coke deposition and, at the same time, to significantly lower the reaction temperature to avoid particle sintering and reduce the operational costs.

Nickel- and cobalt-based catalysts are extensively used in DRM due to their high reactivity and selectivity [17,18,19,20,21]. However, these metals are highly prone to carbon deposition. Noble metals such as Ru, Rh, Ir, etc., although costly, have been found to be much more resilient to carbon formation [12,22,23,24,25,26,27,28,29,30]. The use of metal-oxide supports (TiO_2_, Al_2_O_3_, CeO_2_, etc.) can provide enhanced properties to the catalyst by adjusting the surface area for better dispersion of the metal, thus reducing agglomeration phenomena, and can influence the catalysts resistance to coke formation through alternative reaction pathways [12,15,18,20,23,25,26,27,28,29,30].

Concerning DRM, several studies have suggested a Langmuir–Hinshelwood mechanism where both CH_4_ and CO_2_ are adsorbed on the catalyst surface; however, this pathway tends to promote the formation of graphite [12,23,31,32]. An alternative route is the bifunctional mechanism, also known as Langmuir–Hinshelwood–Hougen–Watson kinetic model, where the support participates in the activation of carbon dioxide and the formation of carbonate species as well as adsorbed oxygen [12,23,32,33,34,35,36]. Methane is adsorbed dissociatively on the catalyst creating adsorbed hydrogen atoms and methyl species. The latter species interact either with the hydroxyl groups or the lattice oxygen of the support producing formates and, eventually, CO on the metal surface. This type of mechanism is mainly proposed for catalyst formulations where the support has intrinsic redox potential enabling the activation of CO_2_. Finally, another study also suggests a direct reaction of gaseous CO_2_ with adsorbed methyl species (Eley–Rideal model) [37]. An in depth analysis of elementary steps considered for the DRM reaction mechanism is presented in [38].

The majority of the studies present in the literature focus on the thermodynamically favorable high-temperature conditions to maximize conversion and selectivity towards the DRM reaction. In contrast, the present study exclusively explores the low temperature regime of the DRM process, where milder operating conditions offer the potential to reduce overall process costs and extend catalyst lifespan. More specifically, this study demonstrates how the appropriate selection of support materials and reactant feed ratios enables precise control of the DRM kinetics, as well as the concurrent RWGS and Boudouard reactions. Notably, high temperature reaction behavior does not necessarily extrapolate to lower temperatures, as the mobility and concentration of oxygen vacancies in redox supports vary as a function of temperature. Ru was selected as an active metal to mitigate carbon deposition, which is thermodynamically favored under the investigated operating conditions, while maintaining a balance between catalyst stability, on-stream activity, and operational cost. Our findings show that redox supports such as TiO_2_ and YSZ (Yttria Stabilized Zirconia, Zr_0.85_Y_0.15_O_1.93_) significantly enhance selectivity towards the DRM reaction and provide improved operational stability compared to other supports. In contrast, non-redox supports like SiO_2_ suffer from rapid deactivation on-stream. Furthermore, the analysis of the CeO_2_-based catalyst highlights the necessity of adjusting the metal particle size to better control its reactivity and on-stream stability and deactivation.

## 2. Results and Discussion

### 2.1. Physicochemical Characterization

Prepared samples were extensively characterized, after the activation treatment, utilizing a number of techniques as mentioned in the experimental section. The catalysts were initially analyzed via X-Ray fluorescence (XRF) to determine the total Ruthenium content (wt%). The results displayed in Table 1 reveal that the YSZ and SiO_2_ supported catalysts exhibit the lowest Ru loading (1.3 wt%), followed by the Ru/CeO_2_ at 2.3 wt%, while Ru/TiO_2_ showed a significantly higher loading (3.7 wt%). These measurements confirm the successful doping of Ru onto the various substrates at distinct loading. Catalysts were prepared with these loadings to enable meaningful comparisons at similar levels of conversion. The total specific area of the catalysts, calculated via N_2_ physisorption, revealed the lowest value for Ru/YSZ at 14 m^2^∙g^−1^. The CeO_2_ and TiO_2_ supported samples have comparable surface areas at ca. 48 m^2^∙g^−1^. In contrast, the Ru/SiO_2_ catalyst exhibited a markedly higher surface area of 282 m^2^∙g^−1^. This discrepancy is expected to contribute to the enhanced activity observed for the Ru/SiO_2_ catalyst, as discussed in later sections.

**Figure 1:**
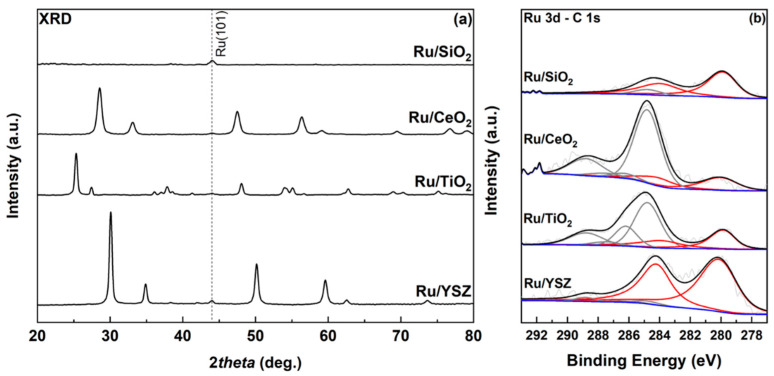
(**a**) XRD spectra of the catalysts under-study in the range of 20 to 80 degrees and (**b**) high resolution XPS spectra of the aforementioned samples in the Ru 3d and C 1s region of 293–277 eV. Red lines correspond to peaks arising from Ru 3d and grey lines to peaks arising from C 1s, in (**b**).

The XRD spectra are displayed in Figure 1a. The presence of ruthenium in the samples is confirmed via the main reflection at 44° attributed to the (101) plane of metallic Ru [39]. In all cases, this peak is significantly weaker than the peaks of the support material, which is attributed to the small quantity of Ru in the samples as well as the small size of the Ru particles. Notably, SiO_2_ appears to have an amorphous structure, as indicated by the absence of any crystalline reflections. The CeO_2_ substrate shows the anticipated peaks at 28.5°, 47.5°, and 56.4°, corresponding to the (111), (220), and (311) planes of the cubic CeO_2_ structure [40]. For the TiO_2_ substrate, the diffractogram shows the expected reflections corresponding to the anatase and the rutile phase of TiO_2_. Quantification of the data showed that the ratio of anatase to rutile phase in the sample is 80:20, in accordance with the ratio provided by the manufacturer. The main peaks for the anatase phase are centered at 25.3°, 48.1°, and 62.7° corresponding to planes (101), (200), and (204), respectively. Rutile phase presence in the sample is confirmed by the reflection at 27.4°, which corresponds to the (110) plane as well as the reflections at 41.2° and 56.6°, which can also be attributed to the rutile (111) and (220) planes [41]. For the Ru/YSZ sample, the Ru(101) peak is once again present at 44.0°. Stabilizing ZrO_2_ with Y_2_O_3_ towards forming YSZ results in a small negative shift of the XRD pattern for ZrO_2_ to slightly smaller diffraction angles. Here, the XRD pattern closely resembles the one expected for YSZ (Zr_0.85_Y_0.15_O_1.93_) [42]. The main peaks of the YSZ substrate are located at 30.1°, 50.2°, and 59.6° for the (111), (220), and (311) planes [43]. Average particle size can be calculated from XRD using the Scherrer equation as shown in the Supplementary Information. The results are summarized in Table 1. Note that the calculation cannot be safely applied for the estimation of the particle size of the Ru/TiO_2_ catalyst, as the Ru(101) peak at 44.0° is distorted by the overlapping (210) signal of rutile TiO_2_.

The samples were further analyzed through X-ray photoelectron spectroscopy (XPS). The Ru 3d area can be seen in Figure 1b. It should be noted that the Ru 3d signal overlaps with the C 1s, requiring careful deconvolution to assess its area and precise position. The 3d_5/2_ peak of Ru is centered at 280.2 eV for Ru/YSZ, 280.0 eV for Ru/CeO_2_, and 279.8 eV for the Ru/TiO_2_ and Ru/SiO_2_ samples. The shifts indicate differing interactions of the metal with the metal oxide support [44], or a different oxidation state. Considering that the Ru 3d_5/2_ peak is expected to center at 280.7 eV for RuO_2_, the first explanation is the likeliest [45]. Specifically, negative shifts in binding energy are expected for stronger interactions of the metal with the support, i.e., the Ru/SiO_2_ and Ru/TiO_2_ catalysts, while they have also been previously reported for TiO_2_ and SiO_2_ substrates [46,47]. In all cases, the spin orbit splitting between the Ru 3d_3/2_ and the Ru 3d_5/2_ is fixed at a value of 4.17 eV as expected from the literature [45,48]. In all samples, Ru appears exclusively in the metallic state. The shape of the Ru 3d_3/2_ peak clearly indicates overlapping contributions from the C 1s region. Fitting of the C 1s contributions was performed according to constraints described in [49], particularly regarding the splitting of the oxygenated carbon species and the main C-C, C-H contribution, which was calibrated to 284.8 eV. Additional components were assigned at 286.2 eV (C-OH functionalities), 287.6 eV for C=O, and 289.0 eV for O-C=O groups [49,50]. For the Ru/SiO_2_ sample, only the C-C peak at 284.8 eV can be resolved from the XPS spectra, most likely due to the sample’s high surface area and the high dispersion of the Ru particles, as will be discussed later, promoting the intensity of the Ru peaks. For the TiO_2_ and CeO_2_ supported catalysts, the main contribution to the total Ru 3d-C 1s spectra belongs to the C-C species due to adventitious carbon, while C-OH and O-C=O groups also show contribution to the overall spectrum. The YSZ supported catalyst shows larger contributions from the Ru peaks, and all C peaks mentioned earlier can be resolved into the fitting, unlike the SiO_2_-based catalyst.

XPS spectra of the O 1s and regions associated with the metals of the supports along with the corresponding analysis can be found in the Supporting Information (Figure S2). The acquired XPS spectra display all the expected oxygen and metal oxide species from the existing literature [51,52,53,54,55,56,57,58,59]. High-resolution scans were also performed in the Cl 2p region (210–190 eV) to detect any residual chlorine ions potentially remaining from the metal precursor. This analysis is particularly important as chlorine is known to act as a catalyst poison and to facilitate coke formation [60,61,62]. Previous studies have shown rapid increase of activity after washing chlorine doped samples with an aqueous or an ammonia solution, which removes the chlorine ions [63]. The only catalyst in which chlorine was observed was Ru/YSZ. However, this does not rule out the possibility of trace amount of chlorine being present in the other samples, below the detection limit of XPS. Overall, the XPS analysis confirmed the presence of the substrate materials in their expected valence states, as well as metallic Ru in all the samples.

Analysis of the Ru XPS spectra in combination with the support metals (Figure S2) allows for the calculation of dispersion, as previously reported for supported catalysts [64]. The dispersion values derived from the XPS data are presented in Table 1, with Ru/SiO_2_ exhibiting the highest value at 81.7%, followed by Ru/YSZ at 40.0%, and lastly the Ru/CeO_2_ and Ru/TiO_2_ samples at 16.7% and 19.2%, respectively. These values are consistent with expectations, as higher metal dispersion corresponds to lower metal loading, as shown from the XRF measurements in Table 1. Using the theoretical dispersion curve for spherical particles (Figure S1), the corresponding particle sizes were calculated and are likewise included in Table 1.

**Figure 2:**
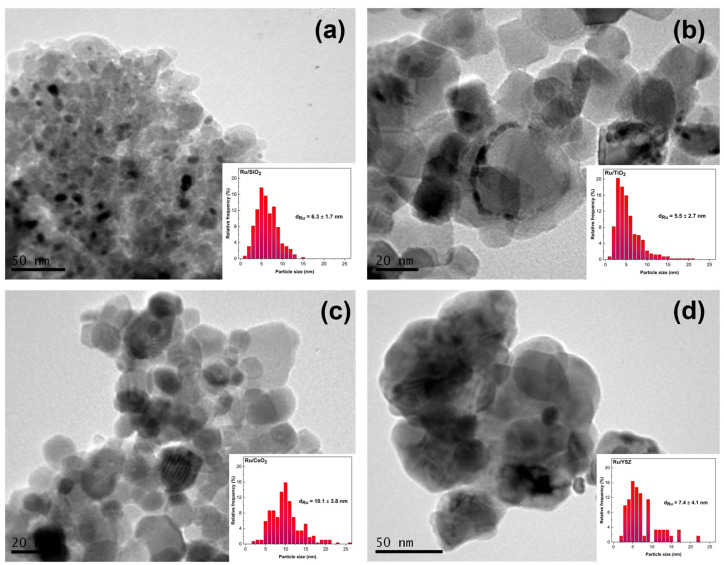
TEM images and corresponding particle size distributions of (**a**) Ru/SiO_2_, (**b**) Ru/CeO_2_, (**c**) Ru/TiO_2_, and (**d**) Ru/YSZ catalyst.

Figure 2 shows selected TEM images and calculated Ru particle size distributions. As shown in Figure 2 and Table 1, the mean Ru nanoparticle size is calculated as 6.3 nm for the Ru/SiO_2_ catalyst, 10.1 nm for the Ru/CeO_2_ sample, 5.5 nm for the Ru/TiO_2_ catalyst, and 7.4 nm for the Ru/YSZ sample. The corresponding standard deviations are 1.7, 3.8, 2.7, and 4.1 nm respectively. Most notably, the TEM images reveal an amorphous support structure in the SiO_2_-based catalyst, consistent with the previously shown XRD pattern. This is indicated by a lack of discrete edges separating the main particles in Figure 2a. The narrower particle size distribution and smaller deviation reflect this observation. In this catalyst, no Ru particles with diameters exceeding 16 nm were observed, unlike the other samples where approximately 2% of the observed particles have a size above 18 nm. It is likely that the large surface area of the SiO_2_ substrate, as confirmed by BET analysis, likely promotes the formation of evenly sized nanoparticles. Furthermore, earlier studies have indicated that broader particle size distributions suggest weaker interactions of the metal with the support surface. Based on this, our results imply that the Ru/TiO_2_ and Ru/SiO_2_ catalysts exhibit the strongest interactions between metal nanoparticles and support among the samples studied [65]. This observation is in excellent agreement with the XPS data reported above. In contrast, the largest particle size deviation is observed for the Ru/YSZ catalyst, which displays the lowest specific surface area as determined by BET measurements. However, this deviation could also be attributed to the lower number of particles sampled, due to the low Z-contrast between Ru and YSZ. The vast majority of the particles in the Ru/TiO_2_, Ru/SiO_2_, and Ru/YSZ sampled fall within the 3–7 nm range, whereas the Ru/CeO_2_ sample shows a broader distribution centered in the 7–12 nm region. This shift is reflected in the higher mean particle size observed for the Ru/CeO_2_ catalyst.

### 2.2. Catalytic Performance

The kinetic measurements presented below were conducted at a flowrate of 200 cm^3^∙min^−1^. This value was selected after studying the catalytic response to an increasing reactant flowrate, while keeping the reactants at a stoichiometric ratio (P_CO2_ = 1 kPa and P_CH4_ = 1 kPa) and the temperature constant. The observed catalytic turnover frequencies (TOFs) and H_2_/CO ratios are presented in Figure S3. It becomes clear that the reaction rate is constant at flowrates above 200 cm^3^∙min^−1^, suggesting that above this value measured and intrinsic rates coincide. Further supporting this claim, the H_2_/CO ratios do not fluctuate much, indicating a constant reaction selectivity with increasing flowrate.

**Figure 3:**
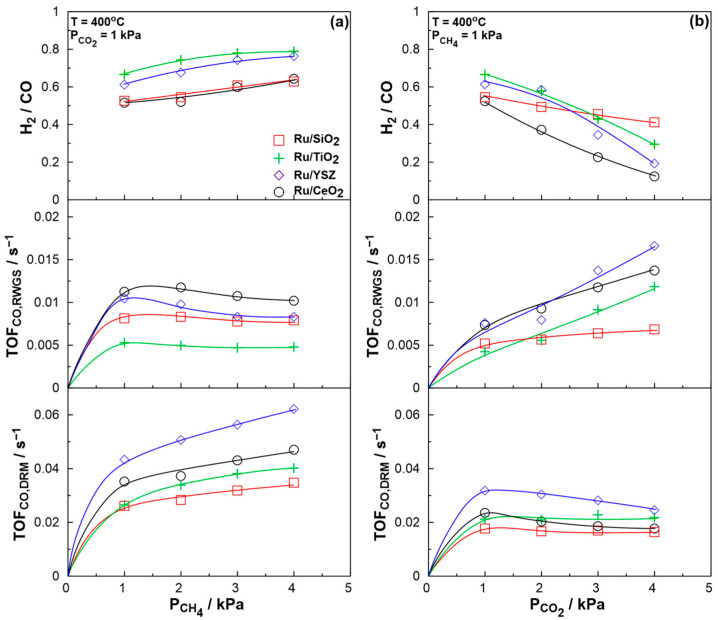
Effect of the reactant ratio on the catalytic rates of production of CO from the DRM reaction (bottom) and the RWGS reaction (middle) and H_2_/CO ratio (top) for (**a**) increasing CH_4_ ratio and (**b**) increasing CO_2_ ratio. Total volumetric flowrate F = 200 cm^3^∙min^−1^.

The effect of reactant ratio on the catalytic rates and H_2_/CO product ratio was initially investigated at 400 °C to ensure, as closely as possible, differential operation conditions. Figure 3 presents the turn-over frequencies of the CO produced from each reaction and the corresponding H_2_/CO product ratio. Increasing the partial pressure of CH_4_, Figure 3a, leads to an increase in the rate of CO production from the DRM reaction, while the CO produced from the RWGS reaction seems to be unaltered or to slightly decrease. This leads to an increase in the H_2_/CO ratio (as seen in the top part of the figure), with maximum values reaching ca. 0.8 for the most reducing conditions applied (P_CO2_ = 1 kPa and P_CH4_ = 4 kPa) when using the Ru/TiO_2_ catalyst. The respective results for increasing CO_2_ partial pressure are displayed in Figure 3b. In this case, shifting to more oxidizing conditions (higher CO_2_/CH_4_ ratio), leads to the opposite effect, which is characterized by an increase in the RWGS reaction rate and a slight reduction in the DRM reaction rate. As previously, this is also evident from the H_2_/CO ratio of the outlet stream, which decreases noticeably with an increase in the CO_2_ partial pressure. It is worth noting that as the conditions become more oxidizing, the Ru/SiO_2_ catalyst, which exhibits the second lowest H_2_/CO ratio under stoichiometric conditions (P_CO2_ = 1 kPa and P_CH4_ = 1 kPa), shifts to the highest H_2_/CO ratio when the most oxidizing conditions are applied (P_CO2_ = 4 kPa). SiO_2_ is generally considered an irreducible support, whereas YSZ, CeO_2_, and TiO_2_ are well known for exhibiting a high concentration of oxygen vacancies, particularly at elevated temperatures. This behavior has been extensively documented through characterization of these materials with methods like Raman spectroscopy and electron paramagnetic resonance (EPR) [66,67,68,69,70,71,72]. These oxygen vacancies are closely associated with the catalytic activity of these materials. During the reaction, under more oxidizing conditions, the scission of the C=O bond in CO_2_ is enhanced, resulting in the formation of adsorbed CO and adsorbed O, promoting the replenishment of the oxygen vacancies with the adsorbed oxygen. These oxygen vacancies have been reported to play a crucial role in the DRM reaction by controlling the CO_2_ activation mechanism on the support through redox cycles [23,73,74]. The catalytic results of the present study demonstrate that, under the most oxidizing conditions tested, the SiO_2_-based catalyst achieves the highest H_2_/CO ratio. This observation implies a different reaction mechanism, where the absence of oxygen vacancies has a lesser impact on selectivity. Earlier studies also indicate that, for a Ru/SiO_2_ catalyst, CO_2_ is more likely to adsorb on the metal rather on the support, which is typically the case for Ru catalysts supported on redox metal oxides [12,23]. It is also observed that increasing the CO_2_ partial pressure has a more significant impact on the H_2_/CO ratio compared to an increase in CH_4_ partial pressure. As shown, the increase in CH_4_ partial pressure promotes the DRM reaction, which is close to its thermodynamic limit at the temperature studied. Consequently, there are no large changes in reaction selectivity despite the noticeable increase in reaction rate.

**Figure 4:**
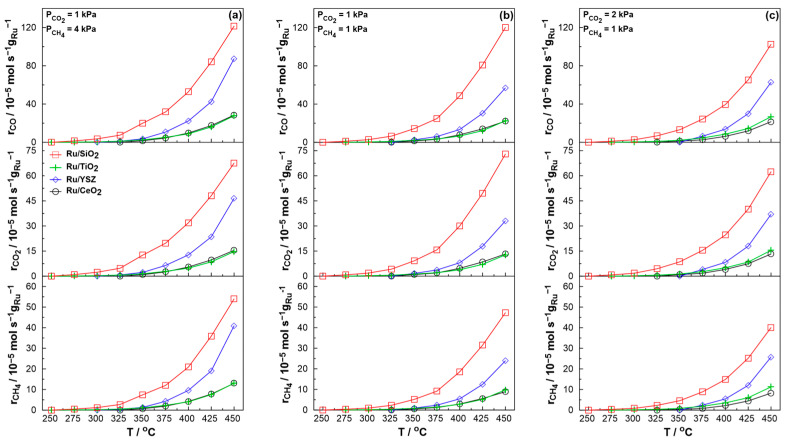
Catalytic rate per Ru mass with increasing temperature for CH_4_ consumption (bottom), CO_2_ consumption (middle), and CO production (top) for (**a**) reducing reaction conditions (P_CO2_ = 1 kPa and P_CH4_ = 4 kPa), (**b**) stoichiometric reaction conditions (P_CO2_ = 1 kPa and P_CH4_ = 1 kPa), and (**c**) oxidizing reaction conditions (P_CO2_ = 2 kPa and P_CH4_ = 1 kPa). Total volumetric flowrate F = 200 cm^3^∙min^−1^.

**Figure 5:**
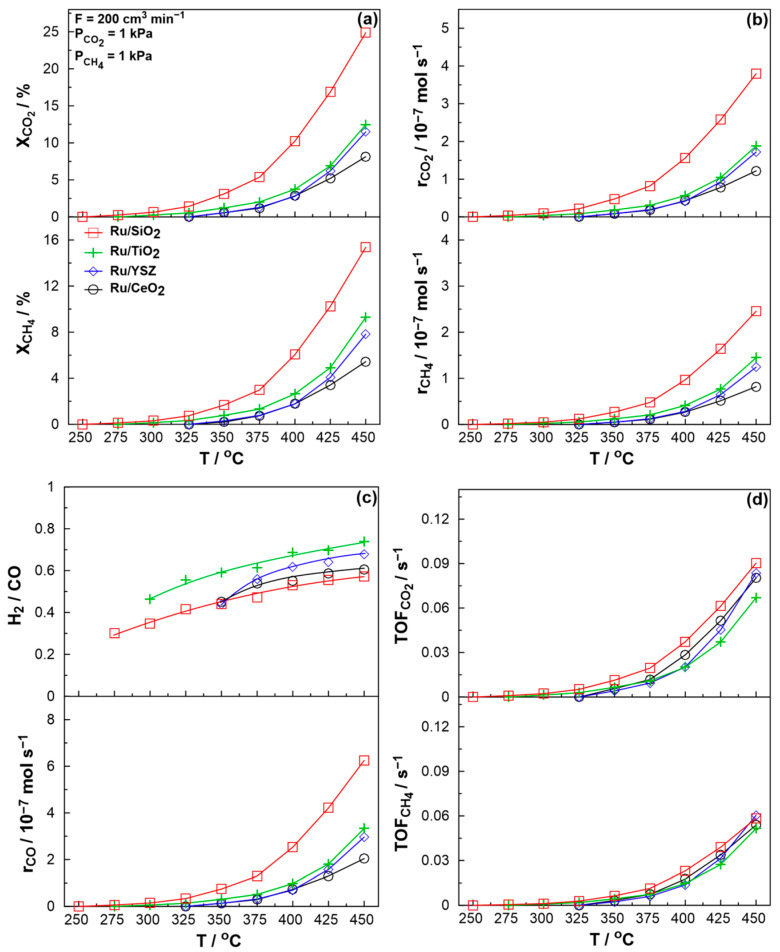
Detailed catalytic results from light-off experiments under stoichiometric conditions (P_CO2_ = 1 kPa and P_CH4_ = 1 kPa). (**a**) Conversion of CH_4_ (below) and CO_2_ (above), (**b**) catalytic rate of reactant consumption for CH_4_ (below) and CO_2_ (above), (**c**) catalytic rate of CO production (below) and H_2_/CO ratio (above), and (**d**) calculated TOFs for CH_4_ (below) and CO_2_ (above). Total volumetric flowrate F = 200 cm^3^∙min^−1^.

Under these reaction temperatures, carbon is deposited on the catalyst’s surface solely through the Boudouard reaction, which involves the C=O bond cleavage (Equation (3)). As the DRM reaction is enhanced with increasing CH_4_ partial pressure, reducing reaction conditions would be expected to be ideal at low temperatures. However, the increase in CO produced through the Dry Reforming of Methane increases the formation of carbon depositing species on the catalyst’s surface. In an effort to identify a balance between deactivation due to carbon deposition and the promotion of the RWGS reaction by increasing the CO_2_/CH_4_ ratio, the reaction system was extensively studied through light-off experiments under three main reaction conditions, namely stoichiometric (P_CO2_ = 1 kPa and P_CH4_ = 1 kPa), reducing (P_CO2_ = 1 kPa and P_CH4_ = 4 kPa), and oxidizing (P_CO2_ = 2 kPa and P_CH4_ = 1 kPa) conditions. Figure 4 illustrates the Ru mass normalized reaction rates achieved under light-off experiments for the catalysts under-study, ranging from reducing to oxidizing conditions (Figure 4a–c). As observed in the figure, reaction rates increase monotonically with temperature due to a combination of thermodynamic and kinetic factors. CO production rates are higher for stoichiometric and reducing conditions, due to the promotion of the DRM reaction compared to the RWGS reaction. Consistent with the earlier kinetic study, under reducing conditions, the CH_4_ consumption rate is enhanced, indicating an increase in the DRM reaction rate and, consequently, the H_2_/CO ratio, across all catalysts. Note that the CO_2_ consumption rate consistently exceeds that of CH_4_, as CO_2_ is consumed in both reactions, whereas CH_4_ is exclusively consumed in the DRM reaction. The highest mass-normalized rates are yielded by the Ru/SiO_2_ catalyst, quite likely due to the catalyst’s large surface area, as seen in Table 1. The sharper increase in the reaction rates for the Ru/YSZ sample yields slightly larger activation energies, as reported later. As oxidizing conditions favor the RWGS reaction and reducing reaction conditions tend to favor deactivation through carbon deposition during initial stability tests, stoichiometric reaction conditions seem to be the optimal scenario for low temperature DRM. At higher temperatures, earlier studies showed a preference for less reducing reaction conditions despite favoring the RWGS reaction, as, above 550 °C, carbon deposition can likely occur through the decomposition of methane [75,76]. Consequently, increasing methane in the inlet stream effectively increases the concentration of carbon depositing species in the reactor. Moreover, stoichiometric and reducing reaction conditions are also more realistic if one considers the use of biogas as an active feedstock for the CO_2_ Reforming of Methane. Given the relative concentrations of CO_2_ and CH_4_ in biogas, the application of reducing and stoichiometric conditions appears to be more cost effective. In this context, catalytic results for stoichiometric conditions are presented in detail in Figure 5.

The catalytic results show that the most active catalyst in terms of raw conversion and reaction rates, Figure 5a,b, is Ru/SiO_2_. This is expected due to the large surface area and high dispersion of the sample. The TiO_2_ and YSZ samples follow, while the lowest activity is observed for the CeO_2_-based catalyst, which also shows the lowest dispersion. In terms of H_2_/CO ratio (Figure 5c, top), the TiO_2_-based catalyst yields the highest values, whereas the SiO_2_- and YSZ-based samples exhibit the lowest values. It is anticipated that Ru/SiO_2_ would show the lowest H_2_/CO ratio, as the current literature suggests, that the ability to form oxygen vacancies (present in all other three substrates) enhances the DRM reaction, thereby shifting the reaction selectivity from RWGS towards DRM [74]. Although the H_2_/CO ratios are constricted between 0 and 1 in the present reaction, significant carbon deposition could actually yield values exceeding 1. This could occur due to the dissociation of CO through the Boudouard reaction and the deposition of carbon on the surface. Therefore, carbon deposition could potentially increase the apparent H_2_/CO ratio, leading to slightly higher observed ratios. When considering that Ru/SiO_2_ exhibits the lowest H_2_/CO ratio but the highest specific activity, one might hypothesize a trade-off between activity and selectivity. However, it is important to note that the surface area of the SiO_2_ substrate is significantly higher than that of the other supports, which likely contributes to the observed increase in the initial reaction rate. Moreover, as reported by Androulakis et al. [12], an irreducible support, Al_2_O_3_, with a surface area closer to those of the other supports studied in their work, showed a catalytic activity at best equal to the least active Ru-based catalysts. This further suggests that observed activity is not solely governed by surface area but rather by the intrinsic properties of the support. The carbon balance, shown in Figure S4 for stoichiometric conditions, converges satisfactorily (less than 1% deviation at the highest temperatures) for the TiO_2_- and YSZ-based samples. In contrast, the Ru/SiO_2_ and Ru/CeO_2_ catalysts display higher deviation values due to surface coking, which also affects the catalysts’ stability, as discussed later. Notably, catalysts based on redox supports show a distinct deviation profile: as temperature increases, the dynamic formation of new vacancies facilitates the partial oxidation of surface carbon species, resulting in a decreasing deviation trend. This behavior contrasts with that of the Ru/SiO_2_ catalyst, where the absence of the carbon removal pathway leads to increasing deviation with temperature, driven by a higher reaction rate.

Figure 5d presents the calculated turn-over frequencies for the reactants under the same conditions. Most notably, the calculated values do not differ significantly, which is indicative of the strong thermodynamic constraints of the reaction system at low temperatures. It is worth noting that, despite having the highest light-off temperature, the Ru/YSZ catalyst shows the fastest activation, yielding the highest CH_4_ TOF at 450 °C, which results in higher activation energies for this catalyst, as described later. The higher CO_2_ TOF for the SiO_2_ supported catalyst results in lower H_2_/CO ratios. Notably, Androulakis et al. [12] recently compared Ru/YSZ and Ru/TiO_2_ catalysts at a slightly higher temperature range and their results are in excellent agreement. H_2_/CO ratios are higher for TiO_2_-based catalysts than YSZ supported catalysts. The specific activity is also higher for Ru/YSZ, in agreement with the results of the present study.

An interesting observation from the light-off experiments was that the YSZ supported catalyst required significantly longer time to achieve a steady state at the light-off temperature. In comparison, the other catalysts achieved conversion almost immediately, once the light-off temperature was attained. We speculate that the formation of an intermediate essential for the reaction to proceed is delayed on the YSZ support, leading to an inevitable delay in the reaction process.

Detailed catalytic results for reducing and oxidizing conditions can be found in Figures S4 and S5. Consistent with the kinetic study presented earlier, the achieved H_2_/CO ratios are higher under reducing conditions. The YSZ supported catalyst stands out in terms of TOF under both oxidizing and reducing conditions, while the SiO_2_ supported catalyst drops to values comparable to the lowest ones. A key difference observed under different conditions is the light-off temperature of the reaction. Specifically, for no catalyst is the light-off temperature lower under oxidizing conditions than reducing. For the SiO_2_- and CeO_2_-based samples, the temperature at which production was first noted remained the same under all three conditions tested. In contrast, for the YSZ-based catalyst, the lowest temperature was observed under reducing conditions, followed by stoichiometric and then oxidizing conditions, indicating that excess methane helps facilitate the onset of the DRM reaction. The TiO_2_-based catalyst showed a light-off temperature of 275 °C for reducing conditions, while this temperature increased to 300 °C for both stoichiometric and oxidizing reactant ratio. The samples that show differences in light-off temperature are known for oxygen vacancies in the support structure at the studied reaction temperatures, therefore explaining these discrepancies. Reducing conditions favor the formation of these vacancies and therefore the activation of the reaction at lower temperatures. The difference noted for CeO_2_ could likely be attributed to the abundance of vacancies in the cerium oxide structure. It is well known that this support exhibits a vast amount of vacancies, even at room temperature. Therefore, the reaction conditions may not promote the formation of new vacancies as effectively as on the other supports, leading to the same light-off temperature under the conditions studied. Another possible explanation for the difference in light-off temperature could simply be the excess of methane in the reaction feed under reducing conditions, as CH_4_ dissociation has been proposed to be the rate determining step of the reaction in previous studies [33,77]. Therefore, increasing CH_4_ concentration would increase the rate of CH_4_ dissociation and therefore, of the reaction.

Calculated activation energies from the light-off experiments can be found in Table 2. It is important to report these values as conducting the reaction at low temperatures enables the observation of the activation phase and, therefore, more reliable activation energy values. The activation energies for each catalyst appear to be largely unaffected by changes in the reactant ratio. The only observable variation is for the RWGS reaction when using the Ru/CeO_2_ catalyst. As the activation energy decreases towards oxidizing conditions, we consider that the CO_2_ dissociation on the support is enhanced, thereby promoting the easier activation of the RWGS reaction when combined with the excess vacancies found in the support structure. One must also note that the RWGS reaction relies on H_2_ as a reactant to produce CO by reacting with CO_2_. Consequently, the reaction requires the prior activation of the DRM reaction, resulting in a reaction system that exhibits hybrid in-series and parallel characteristics. Moreover, as the concentration of CO_2_ exceeds that of H_2_ under the conditions applied in this study, the RWGS reaction takes place under “pseudo-oxidizing” conditions, if one considers CO_2_ as the oxidizing agent and H_2_ as the reducing agent in this context. Additionally, all calculated activation energies fall within the range reported in the current literature [66,76,77,78,79] and are reasonable from a practical point of view. This aligns with observations that lower activation energies are associated with production at lower temperatures, as evidenced by the TiO_2_ and SiO_2_ supported catalyst, which exhibit lower activation energy values.

**Figure 6:**
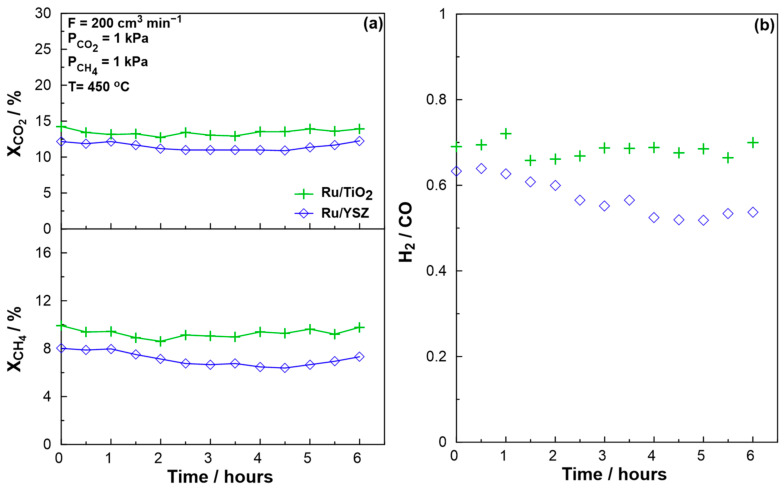
Catalytic performance during a 6 h stability test for selected catalysts under stoichiometric conditions (P_CO2_ = 1 kPa and P_CH4_ = 1 kPa) at 450 °C. (**a**) Reactant conversions over time and (**b**) H_2_/CO ratio evolution over time. Total volumetric flowrate F = 200 cm^3^∙min^−1^.

Selected catalysts were ultimately evaluated for their stability for 6 h under a stoichiometric reaction stream at 450 °C. The results in terms of reactant conversion and H_2_/CO ratio can be seen in Figure 6a and 6b, respectively. The Ru/YSZ catalyst, which showed the highest specific activity towards the DRM reaction, showed no signs of deactivation during the on-stream test. However, the H_2_/CO ratio decreased, implying a shift of the reaction selectivity from DRM towards RWGS. By the 6 h mark, the H_2_/CO ratio had dropped by 10%. A similar trend was reported by Baudouin et al. [80]; however, in that study the decrease in H_2_/CO ratio was accompanied by rapid deactivation of Ni/SiO_2_ catalysts at 500 °C. The SiO_2_-based catalyst showed steep deactivation during an initial 1 h stability test and was therefore not tested over a 6 h period. The underlying reason behind this is the sensitivity of SiO_2_-based catalysts to coke formation, as reported previously [23] and suggested in our case by the non-converging carbon balance. The non-redox properties of SiO_2_ do not allow for oxygen transfer between the support and carbon deposited species/reaction intermediates, therefore coke formation is more likely than catalysts based on redox supports such as CeO_2_ and TiO_2_ [74,81,82,83]. The Ru/TiO_2_ catalyst shows the most satisfactory behavior as both conversion and H_2_/CO ratio appear to be stable under the 6-h experiment. The drawback in this case is that the Ru/TiO_2_ catalyst showed the lowest specific activity towards the reaction. Ru/CeO_2_ was also not tested over a 6-h period as the initial stability tests showed deactivation after only 1 h on stream. Specifically, the ratio CO_1hr_/CO_init._ was equal to 0.78 for the CeO_2_ and 0.89 for the SiO_2_-based catalysts. Conversely, the TiO_2_ and YSZ catalysts were studied for a longer period as these ratios were larger than unity. A study has previously reported carbon deposition as the main deactivation mechanism behind a CeO_2_-based catalyst operating under similar conditions [84]. It also states the possibility of the reducibility of the support yielding an “over reduced” state, where lattice oxygen is not able to further interact with coke species, which in turn enhances carbon deposition. Furthermore, the Ru/CeO_2_ catalyst was found to possess the largest particle size of the studied catalysts. According to the literature, larger metal particle sizes promote coke formation on the catalytic surface, leading to deactivation on stream. Our catalytic results show that even at ca. 10 nm particle size, Ru/CeO_2_ deactivates rapidly after 1 h on-stream, despite its excellent redox properties and the noble nature of the active metal. In turn, careful tuning of the catalyst particle size is required for the application of the DRM process at low temperatures, as the Ru/CeO_2_ catalyst, one of the most active and stable under high temperature conditions, shows rapid deactivation under low temperature DRM [24,85,86,87]. We propose that the slightly stronger metal-support interactions for the Ru/TiO_2_ catalyst, accompanied by the redox properties of the support phase, strongly enhance the coke deposition resistance.

**Figure 7:**
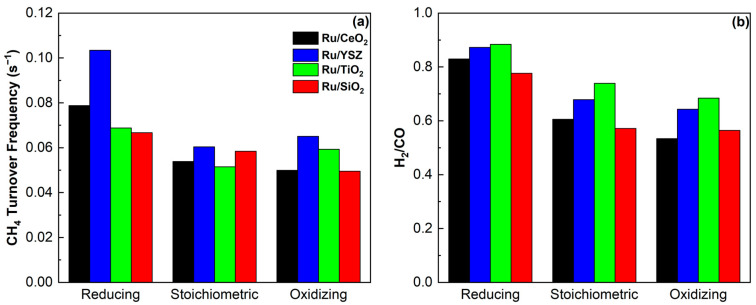
(**a**) Methane turnover frequency and (**b**) H_2_/CO ratio for all light-off experiments at 450 °C.

Total comparative results of the light-off experiments are shown in Figure 7. The CH_4_ TOF, presented in Figure 7a, is enhanced under reducing conditions, in agreement with the earlier kinetic study. Furthermore, under all reaction conditions, the YSZ supported catalyst shows the highest values, despite the highest light-off temperature as reported earlier. The TiO_2_ supported catalyst presents the lowest CH_4_ TOF under stoichiometric conditions, while the SiO_2_ supported catalyst shows the lowest values under the other two conditions studied. This decline of the catalytic activity of Ru/SiO_2_ can likely be attributed to the redox potential and the acid-base properties of the other supports, which enable better adaptability to both reducing and oxidizing conditions. SiO_2_ on the other hand is mildly acidic and therefore does not interact strongly with CO_2_ [88,89,90]. As a result, the increase in partial pressure has a much smaller effect in this case. The TiO_2_ and YSZ supported catalysts also show a small increase in CH_4_ TOF when moving from stoichiometric to oxidizing conditions. The H_2_/CO ratios, seen in Figure 7b, confirm the promotion of the DRM reaction under reducing conditions. Higher values are achieved for the Ru/TiO_2_ catalyst under all reaction conditions; however, this gap closes under reducing conditions. Once again, a different behavior is noted for the SiO_2_ supported catalyst, which shifts to higher ratios than Ru/CeO_2_ under oxidizing conditions. As mentioned above, SiO_2_ is an irreducible support, therefore a different reaction mechanism is likely expected. This is consistent with the literature [91], where the increase in the partial pressure of CO_2_ does not significantly impact reaction selectivity. During the kinetic study presented above, it was shown that the addition of larger quantities of CO_2_ to the feed has a much lesser effect on Ru/SiO_2_ than on the other catalysts.

Despite a study claiming that activity and selectivity of the reaction system are independent of metal particle size [80], most studies in the existing literature cite the increase of nanoparticle size as a reasoning for shift of reaction selectivity towards the RWGS reaction and, consequently, a decrease in the H_2_/CO ratio [92,93,94]. Under stoichiometric and oxidizing conditions, the H_2_/CO ratio for the Ru/CeO_2_ catalyst approaches that of the Ru/SiO_2_ catalyst more compared to the YSZ and TiO_2_ supported catalysts. Evidently, the increased Ru particle size likely explains this observation.

## 3. Experimental

### 3.1. Catalyst Preparation

The under-study catalysts were prepared using a simple wet impregnation method. Specifically, the required amount of the metal precursor salt RuCl_3_·3H_2_O (Ventron Chemicals, Mumbai, India) was dissolved in 10 mL of deionized water and then added to a solution containing the required amount of substrate powder and 30 mL of distilled water under rigorous stirring. Four catalyst substrates were utilized in powder form: YSZ (Tosoh Corporation, Tokyo, Japan), TiO_2_ P25 (Degussa, Frankfurt, Germany), CeO_2_ (Alfa Aesar, Ward Hill, MA, USA), and SiO_2_ (Sigma Aldrich, St. Louis, MO, USA). The mixture was then magnetically stirred for 20 min at room temperature, 20 min at 50 °C, and then heated to 80 °C until complete water evaporation. The resulting catalyst paste was dried overnight at 80 °C and then calcined at 500 °C for 2 h following a 10 °C∙min^−1^ heating rate. Catalyst powder was then sieved and only particles under a 0.25 mm diameter were used in the catalytic study. Prior to any catalytic experiments, the catalyst was activated under a 100 cm^3^∙min^−1^ flowrate of 15% H_2_/He (Linde, Dublin, Ireland) at 400 °C for 2 h, in order to fully reduce formed RuO_2_ to metallic Ru [95]. Different loadings of Ru were applied to the various substrates in order to achieve comparable levels of conversions across the catalysts.

### 3.2. Physicochemical Characterization

The physicochemical properties of the synthesized materials were assessed utilizing a range of techniques including X-ray diffraction (XRD), X-ray photoelectron spectroscopy (XPS), X-ray fluorescence (XRF), N_2_ physisoprtion (BET) analysis, and transmission electron microscopy (TEM).

X-ray fluorescence spectroscopy (XRF) measurements were carried out using a benchtop Energy-Dispersive X-Ray fluorescence spectrometer (S2 PUMA, Bruker Nano GmbH, Berlin, Germany). X-ray diffraction (XRD) experiments took place using a Bruker D8 Advance X-ray diffractometer, equipped with Ni-filtered Cu-Kα radiation (λ = 0.154 nm). Scans were collected in the 2θ interval of 20–80°, with a scanning step of 0.015°. Samples were further morphologically analyzed through TEM imaging, utilizing a JEOL (Tokyo, Japan) 2010 electron microscope with a maximum acceleration voltage of 200 kV. Various images, which were captured using an Erlangshen CCD Camera (Gatan Model 782 ES500W, Pleasanton, CA, USA) at differing magnifications were used in order to extract the mean Ru particle size and the standard deviation.

N_2_ physisorption was performed on all samples in a Quantachrome iQ series apparatus. For the experiments 100–200 mg of samples were degassed at 120 °C under vacuum for 2 h and then re-weighed before the measurements of N_2_ adsorption at 77 K. The BET equation was applied in the range of 0.05–0.3 P/P_0_ to calculate the specific surface area (SSA).

X-ray photoelectron spectroscopy (XPS) measurements were carried out in an ultra-high vacuum system, which has been extensively described in previous work [96,97]. Scans were recorded using an electron energy analyzer (Leybold LH EA11, Cologne, Germany) operated at a pass energy of 100 eV and a non-monochromatic AlKα line at 1486.6 eV. High resolution scans in areas of interest were recorded at 0.1 eV intervals and all scans were charge corrected with reference to adventitious carbon at 284.8 eV. Samples were prepared by depositing the catalyst specimen on a thin lead sheet. Data analysis took place with the XPS PEAK 4.1 software, applying a Shirley background. The Ru 3d region was deconvoluted according to the method reported by Morgan et al. [45]. Contributions from the C 1s region were fitted with constraints based on the expected splitting of oxygenated carbon species from the main C-C, C-H peak, which was calibrated to 284.8 eV to account for adventitious carbon [49]. No constraints were applied between the set of carbon peaks and those attributed to ruthenium. The calculation of dispersion from XPS data is performed as reported in [64] and is briefly explained in the Supporting Information. The surface sensitivity of a technique such as XPS, provides the necessary information for dispersion to be calculated via the model first proposed by Kerkhof et al. [98], with an error of 10% of the calculated value [64,99,100,101,102,103]. The subsequent calculation of mean particle size, considering spherical particle shape is performed utilizing the theoretical dispersion curve for Ru metal, illustrated in Figure S1. The procedure followed towards the creation of this curve is also explained in the Supporting Information. All material characterization was carried out after the activation treatment described in Section 3.3.

### 3.3. Catalytic Performance Evaluation

Catalytic experiments took place within a tubular quartz fixed-bed reactor with an outer diameter of 6 mm and an inner diameter of 3 mm. Inert quartz wool was used to keep the catalyst bed (40 mg of powder) in place at the center of the reactor, which was then fixed in a tubular furnace. The temperature of the catalyst bed was monitored using a K-type thermocouple. CO_2_ (25% in He), H_2_ (15% in He), CH_4_ (5% in He), and He (99.999%) were acquired from Linde. Flowrates from each vessel could be controlled via electronic flowmeters (Brooks Electronics, Chelmsford, MA, USA). The total volumetric flowrate was kept constant at 200 cm^3^∙min^−1^, unless otherwise stated. The outlet stream from the reactor was analyzed by an infrared gas analyzer (Fuji Electric, Tokyo, Japan) and a gas chromatograph (Shimadzu, Kyoto, Japan) using a thermal conductivity detector (TCD) and a flame ionization detector (FID). During catalytic experiments, the temperature was raised from 200 °C to 450 °C with a 25 °C step until a steady state was observed. The reactor was subsequently cooled under 200 cm^3^∙min^−1^ under He flow to room temperature. Light-off experiments were performed at three different reactant ratios, oxidizing reaction conditions (P_CO2_ = 2 kPa and P_CH4_ = 1 kPa), stoichiometric reaction conditions (P_CO2_ = 1 kPa and P_CH4_ = 1 kPa), and reducing reaction conditions (P_CO2_ = 1 kPa and P_CH4_ = 4 kPa). No other product, other than those mentioned in the introduction (H_2_, CO and H_2_O), was detected. The kinetic study was performed at a constant flowrate of 200 cm^3^∙min^−1^ under eight reactant ratios and a temperature of 400 °C. The reactant ratios were intentionally kept relatively dilute to avoid carbon deposition, which is thermodynamically favored under low temperature conditions. It has been previously shown that combining dilute feed ratios and a noble metal/metal oxide catalytic system can enhance the systems resistance to carbon deposition [75,104]. The catalyst powder was reduced in situ before every experiment for 1 h under a 200 cm^3^∙min^−1^ flow of 15% H_2_/He. The extensive protocol behind the catalytic calculations can be found in the Supporting Information. Specifically, the calculation of turn-over frequencies (TOFs) is made according to Equation (5).

(5) $$TOF\;\left[s^{-1}\right]=r\left[\frac{mol}{g_{cat}s}\right]\cdot \frac{AW_{Ru}}{D\cdot M}\;\;$$

where D is the dispersion calculated via XPS and M is the weight loading of Ru in the sample as determined through X-ray fluorescence (XRF).

Catalysts are further compared on a TOF basis as the addition of active metal sites is the activating force behind the reaction [105]. All catalytic experiments were carried out on fresh catalyst powder that had undergone activation treatment, followed by 1 h in situ reduction as described above.

## 4. Conclusions

The present study demonstrates the feasibility of low-temperature Dry Reforming of Methane (DRM) as a cost-effective alternative to conventional high-temperature processes. The achieved H_2_/CO ratios are remarkably high for the studied temperature range, particularly under reducing conditions that favor DRM over the Reverse Water Gas Shift (RWGS) reaction. The application of the DRM reaction at these temperatures is intriguing as it reduces running costs by a significant margin. The challenging thermodynamics of the reaction make it difficult to achieve competitive conversions at low temperatures while also managing coke deposition. However, a well-optimized noble metal/redox support catalytic system can ensure stability under these conditions. Redox supports, such as TiO_2_ and YSZ, increase the H_2_/CO ratio, indicating a shift in reaction selectivity toward DRM. In contrast, Ru/SiO_2_ and Ru/CeO_2_ catalysts exhibited deactivation due to coking, while YSZ and TiO_2_ supported catalysts maintained stable on stream reactivity; however, the YSZ supported catalyst displayed a shift of reaction selectivity to the RWGS reaction. The Ru/TiO_2_ catalyst is the most promising of the under-study catalysts, as, despite the lowest specific activity, it shows the highest selectivity towards DRM, coupled with the best overall performance in the stability test. These results underscore the key influence of the support material’s physicochemical properties in directing the DRM process. Furthermore, our results highlight the importance of tuning metal particle size, especially at low temperatures. Notably, smaller metal nanoparticles effectively mitigated coking, while carbon deposition remained the primary cause of catalyst deactivation. The calculated activation energies ranged from 24–33 kcal∙mol^−1^ for DRM and 19–30 kcal∙mol^−1^ for RWGS. Future research should prioritize low-temperature DRM and explore alternative strategies to enhance activity and the H_2_/CO ratio under mild operating conditions. The extraction of further activity out of Ru/TiO_2_ supported catalysts can provide high yields towards DRM, while maintaining the stability of the catalyst under operating conditions.

## Figures and Tables

**Table 1:** Physicochemical properties of the under-study catalysts prepared as described in Section 3.1.

Sample	Ru wt% ^1^	SSA ^2^ (m^2^ g^−1^)	Ru Particle Size ^3^ (nm)	Ru Particle Size ^4^ (nm)	Ru Dispersion ^5^ %	Ru Particle Size ^6^ (nm)
Ru/YSZ	1.3	14.4	7.4 ± 4.1	14.9	40.0	2.7
Ru/CeO_2_	2.3	48.9	10.1 ± 3.8	12.3	16.7	6.2
Ru/TiO_2_	3.7	46.8	5.5 ± 2.7	n.a. ^7^	19.2	5.5
Ru/SiO_2_	1.3	281.7	6.3 ± 1.7	9.1	81.7	1.2

^1^ Determined through XRF. ^2^ Determined through N_2_ physisorption. ^3^ Average estimated through TEM. ^4^ Calculated with Scherrer’s equation through XRD. ^5^ Determined through XPS. ^6^ Determined through XPS, assuming spherical particles. ^7^ Not applicable.

**Table 2:** Estimated activation energies (kcal∙mol^−1^) under differing reaction conditions for the two reactions.

Catalyst	Reaction	Reducing	Stoichiometric	Oxidizing
Ru/YSZ	DRM	33 ± 2	30 ± 1	29 ± 1
	RWGS	24 ± 3	24 ± 2	24 ± 2
Ru/CeO_2_	DRM	28 ± 2	28 ± 1	30 ± 2
	RWGS	30 ± 3	24 ± 2	20 ± 2
Ru/TiO_2_	DRM	24 ± 1	23 ± 2	26 ± 1
	RWGS	21 ± 1	19 ± 1	21 ± 1
Ru/SiO_2_	DRM	25 ± 2	24 ± 1	24 ± 1
	RWGS	22 ± 2	21 ± 1	22 ± 1

## Data Availability

Data will be made available upon reasonable request to the corresponding author.

## Supplementary Materials

The following supporting information can be downloaded at: https://www.mdpi.com/article/10.3390/molecules30102135/s1. Refs. [51,52,53,54,55,56,57,58,59,64,98,102,103] are cited in the Supplementary Materials.
