# Supplementary material for: CO2 Reforming of Methane over Ru Supported Catalysts Under Mild Conditions
Source: Molecules. 2025 May 12;30(10):2135. doi: 10.3390/molecules30102135 (PMC12114345; doi:10.3390/molecules30102135)
Supplement: Supplementary file 1 [file molecules-30-02135-s001.zip › molecules-3596048-supplementary.pdf]

SUPPORTING INFORMATION

CO_2 Reforming of Methane over Ru supported catalysts under mild conditions

Alexandros K. Bikogiannakis[1], Andriana Lymperi[1], Paraskevas Dimitropoulos[1,2], Kyriakos Bourikas[3], Alexandros Katsaounis[1], Georgios Kyriakou[1,*]

1 Department of Chemical Engineering, University of Patras, 26504, Patras, Greece; bikos@chemeng.upatras.gr (A. B.); lymperi@chemeng.upatras.gr (A. L.); alex.katsaounis@chemeng.upatras.gr (A. K.), parisdim@iceht.forth.gr (P. D.)
2 Institute of Chemical Engineering Sciences, Foundation for Research and Technology, 26504, Patras, Greece;
3 School of Science and Technology, Hellenic Open University, 26335, Patras, Greece; bourikas@eap.gr
* Correspondence: kyriakg@upatras.gr

Calculation of dispersion form XPS data.

Dispersion is calculated as reported in [1], applying a simplified version of the model first reported by Kerkhof et al. [2]. Briefly, the model compares the values of theoretic and experimental ratios of the XPS intensities for the active phase metal and the metal of the support, considering sheets of the support and the photoexcited electrons of the active metal travelling through those sheets. The thickness of the aforementioned sheets is estimated through equation (S1), in which S_s is the specific surface area of the support and ρ_s is the support density.

$$t = \frac{2}{S_s \rho_s} \quad (S1)$$

The dimensionless parameter β then compares the thickness of these sheets with the inelastic mean free path of an electron excited from the $3d_{5/2}$ bound state of Ruthenium (kinetic energy equal to ca. 1207 eV when excited by an AlKα source, 1486.6 eV) travelling through the support sheets (λ) (equation (S2)). The inelastic mean free path was calculated through the Tanuma-Powell-Penn 2M model (TPP2M) [3].

$$\beta = \frac{t}{\lambda} \quad (S2)$$

The theoretical ratio is given by equation (S3), where at_m and at_s symbolize the atomic ratios of the active metal and the support metal (most commonly part of the metal oxide).

$$\left(\frac{I_m}{I_s}\right)_{th} = \left(\frac{at_m}{at_s}\right)\frac{\sigma_m}{\sigma_s}\frac{\beta}{2}\frac{(1+e^{-\beta})}{(1-e^{-\beta})} \quad (S3)$$

In the above equation, σ_m and σ_s symbolize the Scofield cross sections of the excited photoelectrons as derived from [4] for AlKα rays (1486.6 eV).

The values of the IMFP and Scofield cross sections are reported below in Table S1.

Table S1. Parameters used towards the calculation of dispersion via XPS.

Metal	Ru	Ti	Zr	Si	Ce
Peak	$3d_{5/2}$	$2p_{3/2}$	$3d_{5/2}$	$2p_{3/2}$	$3d_{5/2}$
Scofield Cross Section σ	7.39	5.22	4.17	0.541	30.5
Inelastic Mean Free Path (Å)	n.a.	24.04	21.52	28.00	19.77

The dispersion of the catalysts is finally calculated by comparing the value of the experimental ratio of the intensity of the XPS peaks named in Table S1 through equation (S4).

$$D = \frac{\left(\frac{I_m}{I_s}\right)_{exp}}{\left(\frac{I_m}{I_s}\right)_{th}} \cdot 100\% \quad (S4)$$

Catalytic calculations protocol.

Conversions of each reactant are calculated using the following equation:

$$X[\text{react.}] = \frac{[\text{react.}]_{in} - [\text{react.}]_{out}}{[\text{react.}]_{in}} \quad (S5)$$

Catalytic rates of consumption or production under differential conditions (maximum conversion applied 20%) are calculated by equation (S6):

$$r[\text{react. or prod.}] = ppm_{\text{react. or prod.}} \frac{F_V}{V_{molar}} \quad (S6)$$

Where, F_v is the volumetric flowrate of the reacting mixture and V_{molar} is the molar volume under STP conditions, considered 22400 $cm^3 \cdot mol^{-1}$.

Rates of each reaction were estimated through the following equations:

$$r_{CO,DRM} = 2r_{CH_4} \text{ (S7)}$$

$$r_{CO,RWGS} = r_{CO_2} - r_{CH_4} \text{ (S8)}$$

Activation energies of the reactions are calculated via the Arrhenius equation (S9).

$$\ln r = -\frac{E_a}{RT} + C \text{ (S9)}$$

Their calculation is performed graphically applying equation (S9) to the first 4 or 5 points of the plot.

Turnover frequency is calculated in units of inverse seconds applying equation (S10).

$$TOF\ [s^{-1}] = r\left[\frac{mol}{g_{cat}s}\right] \cdot \frac{AW_{Ru}}{D \cdot M} \text{ (10)}$$

Where D is the dispersion calculated via XPS as described above, and M is the weight loading of Ru in the sample as determined through X-ray Fluorescence (XRF).

The % deviation in the carbon balance is calculated by equation (S11).

$$\%\ deviation = \frac{|[CO_2]_{in} + [CH_4]_{in} - [CO]_{out} - [CO_2]_{out} - [CH_4]_{out}|}{[CO_2]_{in} + [CH_4]_{in}} \text{ (S11)}$$

Scherrer's equation.

Scherrer's equation allows for the estimation of particle sizes from X-ray diffraction peak data. Specifically, through equation (S12) the mean crystallite size can be estimated.

$$d = \frac{K \cdot \lambda}{B(2\theta) \cdot \cos\theta} \text{ (S12)}$$

In the above equation,

d is the mean crystallite size

K is shape factor, considered approximately 0.9 for spherical particles

λ is the wavelength of the incident radiation (0.1504 nm)

B(2θ) is the full width at half maximum of the peak the equation is being applied to, and

cosθ is the cosine of the half of the diffraction angle.

Supporting data.

Figure S1 shows the theoretical dispersion curve as a function of Ruthenium particle size, when considering the particles spherical. Starting from a known particle diameter, the surface atoms are calculated from the surface of the sphere and the area a Ruthenium atom occupies on the surface (cross sectional area, $8.6 \cdot 10^{-16}$ $cm^2 \cdot atom^{-1}$), which can be found in the main text. The bulk Ruthenium atoms can be calculated considering the bulk radius of the particle as the original radius minus the diameter of a Ruthenium atom (1.34 Å). The bulk radius then yields a bulk Ruthenium volume, which can then be treated in two ways. Either considering the Ru density (12.3 $g \cdot cm^{-3}$), and molar mass (101.07 $g \cdot mol^{-1}$), thus yielding the bulk atoms, or calculating the volume of a unit cell of the Ruthenium HCP structure (81.7 $Å^3$) and considering 6 atoms per HCP unit cell deriving the total number of bulk atoms. This amount is then added to the surface atoms, yielding the total Ru atoms and thus, dispersion. The two curves have a maximum offset of 0.1% in the area presented.

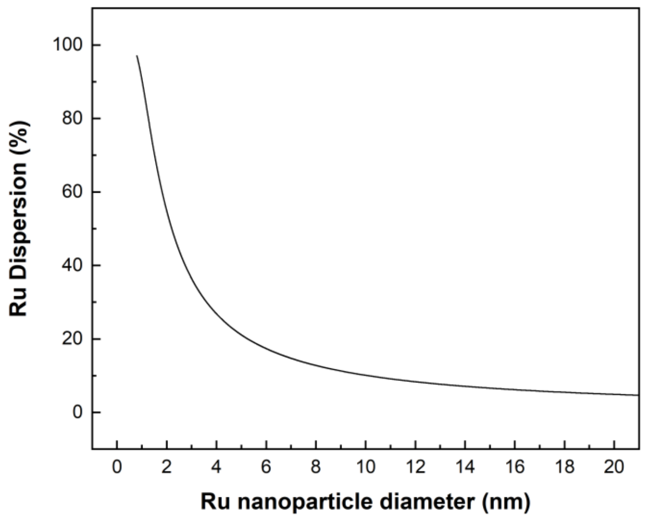

Figure S1. Theoretic curve of dispersion as a function of Ru particle size considering spherical shape.

The O 1s spectra can be observed in Figure S2(a). The O 1s peak centers at 529.6 eV for CeO_2, 530.0 eV for TiO_2 and 532.9 eV for SiO_2, all values that have been previously reported for each oxide respectively [5–7]. Shoulders at higher binding energies can be observed for CeO_2 and TiO_2 due to the formation of surface -OH groups, possibly due to the interaction of the surface with background hydrogen gas present in the UHV chamber. This shoulder isn't observed for SiO_2. On the other hand, the YSZ sample shows a main feature centered at 529.8 eV, while a shoulder for surface -OH formalities is also observed. In this case, a shoulder is also observed at lower binding energies, likely due to the interactions of oxygen with yttrium in the oxide (Y_2O_3) lattice. For the aforementioned sample, the Zr $3d_{5/2}$ peak centers at 182.1 eV, with its $3d_{3/2}$ doublet at 184.5 eV, with a 3.3 eV splitting, confirming the +4 oxidation state in the sample, as seen in Figure S2(b). Likewise, in Figure S2(c), the Y 3d peak shows contributions from the $d_{5/2}$ and $d_{3/2}$ states at 157.1 eV and 159.2 eV respectively, confirming the +3 oxidation state for yttrium [8–10]. For the Ru/TiO_2 catalyst, the Ti 2p region (Figure S2(d)) shows contributions from the main photoemission peak of Ti $2p_{3/2}$ at 458.7 eV, while the $2p_{1/2}$ doublet centers at 464.4 eV. The positions of these peaks, along with the 5.7 eV splitting of the two 2p states, are characteristic of Ti in the 4+ oxidation state, as expected for TiO_2 [5,11]. The spectrum also contains a contribution from the Ru $3p_{3/2}$ peak at 461.0 eV, for metallic Ru. Finally, a satellite peak for TiO_2 is observed at binding energies ca. 472 eV. Figure S2(e) depicts the Ce 3d spectrum, as recorded for the Ru/CeO_2 catalyst. The complex structure shows strong peaks at 882.9 eV for the main Ce $3d_{5/2}$ peak and secondary peaks at 888.9 eV and 898.5 eV. The $3d_{3/2}$ component of the spectrum yields contributions at 901.2 eV, with secondary peaks arising at 907.2 eV and 917.0 eV. The secondary peaks arise due to final state effects, as claimed by Beche et al. [12] and Morgan [7]. The highest binding energy states for each component of the 3d spectra (the peaks at 898.5 and 917.0 eV) arise only for Ce in the +4 oxidation state, therefore confirming the presence of CeO_2 in the sample. Finally, the Si 2p peaks for the Ru/SiO_2 catalyst can be seen in Figure S2(f). The doublet consists of two strong peaks at 103.4 eV and 104.0 eV for $2p_{3/2}$ and $2p_{1/2}$, as previously reported for Si in the +4 oxidation state [13].

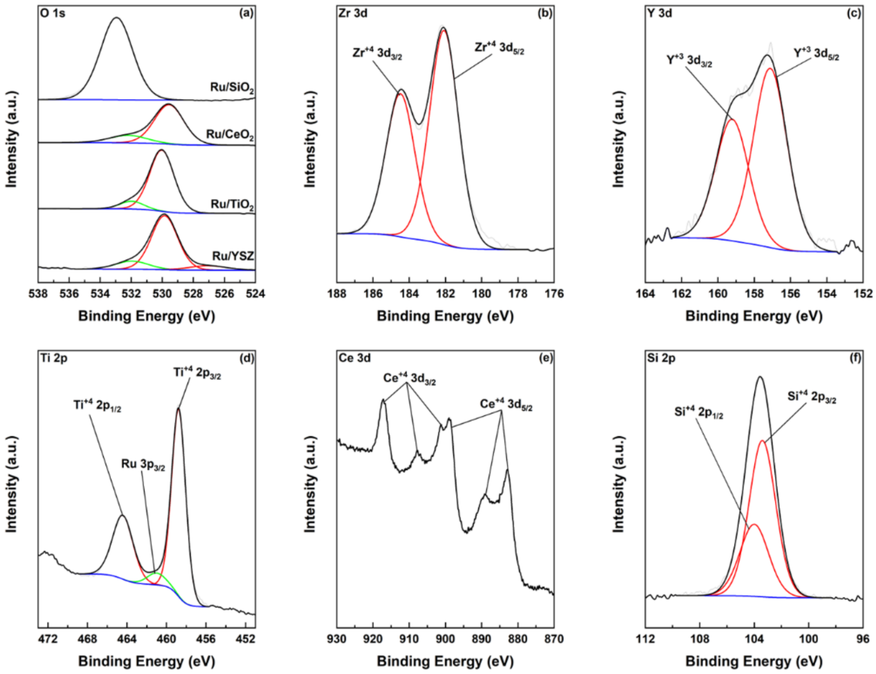

Figure S2. High resolution XPS spectra of the regions of interest for the under study catalysts. (a) O 1s, (b) Zr 3d, (c) Y 3d, (d) Ti 2p, (e) Ce 3d and (f) Si 2p.

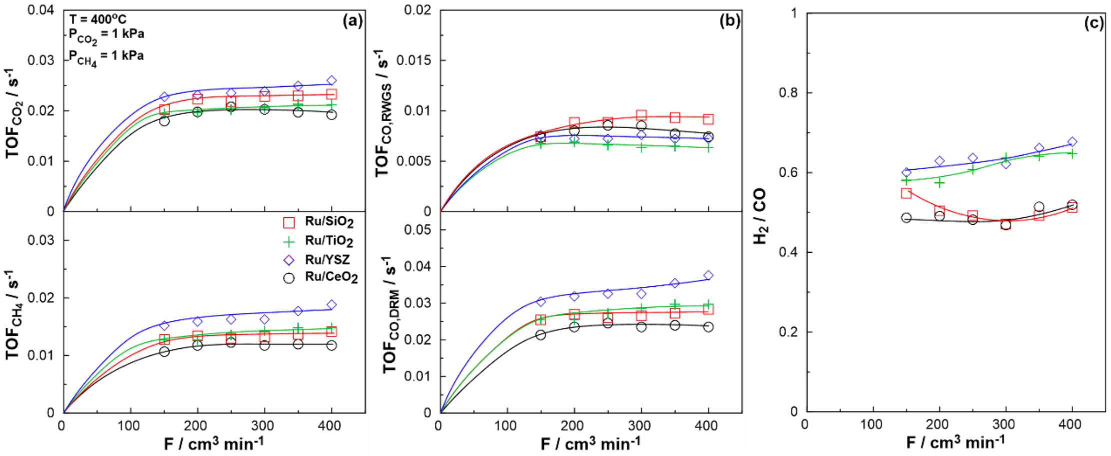

Figure S3. Catalytic rate dependence on the reactant mixture volumetric flowrate for (a) CH_4 consumption (bottom) and CO_2 consumption (top), (b) CO production via the DRM reaction (below) and CO production via the RWGS reaction (above) and (c) H_2/CO ratio.

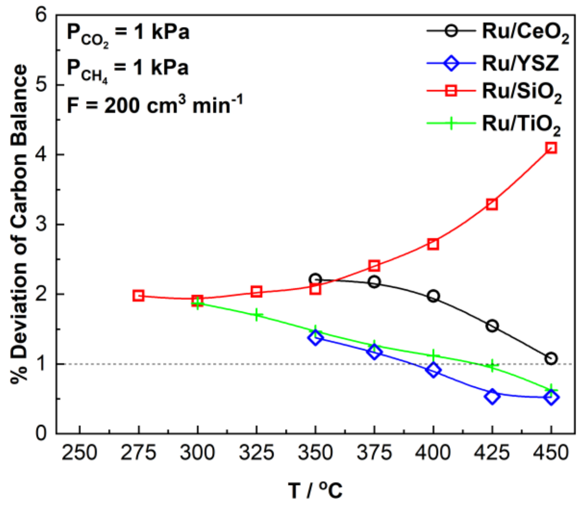

Figure S4. % Deviation of the carbon balance as a function of temperature for the light-off experiments under stoichiometric conditions (P_{CO_2} = 1 kPa and P_{CH_4} = 4 kPa).

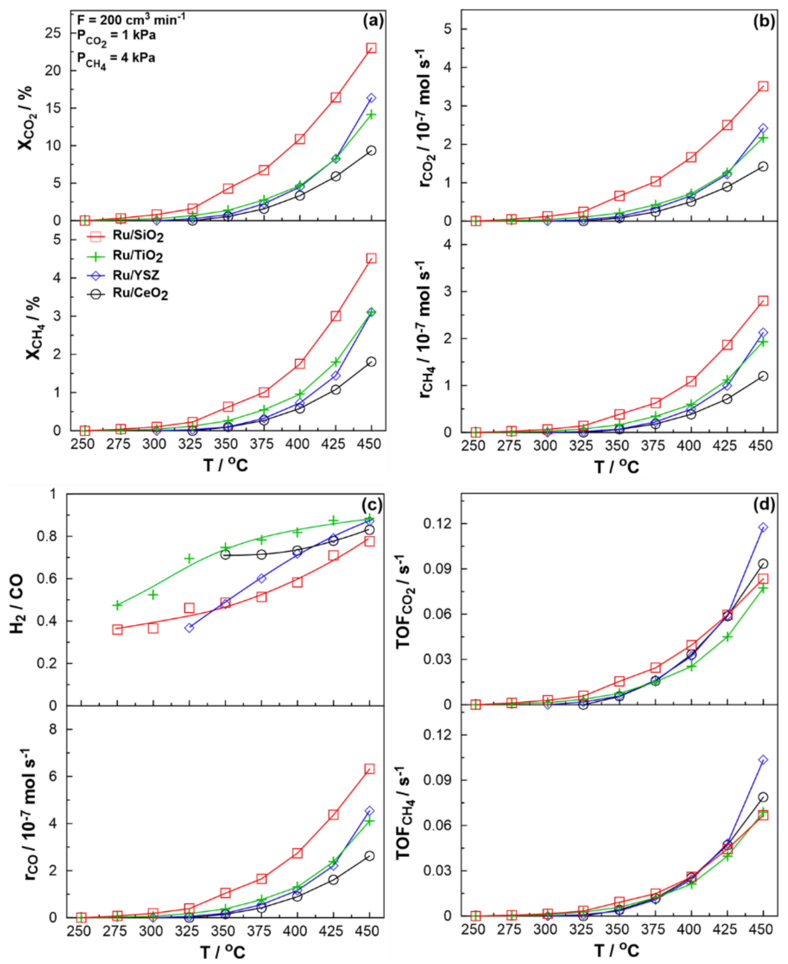

Figure S5. In detail catalytic results for light-off experiments under reducing conditions (P_{CO_2} = 1 kPa and P_{CH_4} = 4 kPa). (a) Conversion of CH_4 (below) and CO_2 (above), (b) Catalytic rate of reactant consumption for CH_4 (below) and CO_2 (above), (c) Catalytic rate of CO production (below) and H_2/CO ratio (above), and (d) Calculated TOFs for CH_4 (below) and CO_2 (above).

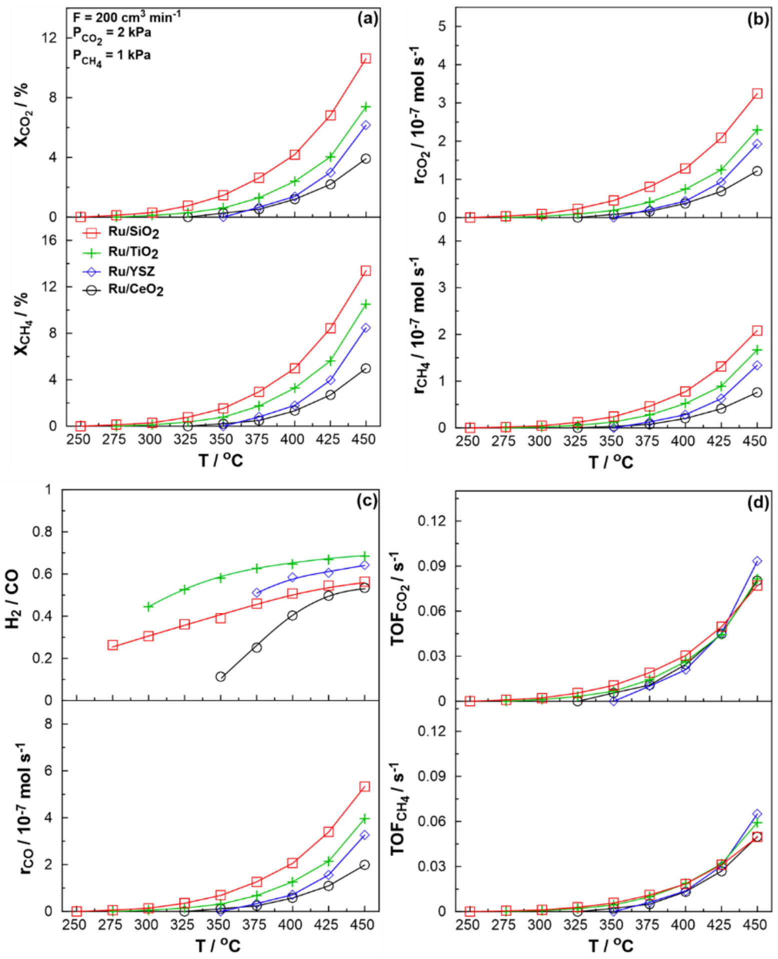

Figure S6. In detail catalytic results for light-off experiments under oxidizing conditions (P_{CO_2} = 2 kPa and P_{CH_4} = 1 kPa). (a) Conversion of CH_4 (below) and CO_2 (above), (b) Catalytic rate of reactant consumption for CH_4 (below) and CO_2 (above), (c) Catalytic rate of CO production (below) and H_2/CO ratio (above), and (d) Calculated TOFs for CH_4 (below) and CO_2 (above).

References

1. Islam, M.J.; Granollers Mesa, M.; Osatiashtiani, A.; Manayil, J.C.; Isaacs, M.A.; Taylor, M.J.; Tsatsos, S.; Kyriakou, G. PdCu Single Atom Alloys Supported on Alumina for the Selective Hydrogenation of Furfural. Appl. Catal. B Environ. **2021**, 299, 120652, doi:10.1016/j.apcatb.2021.120652.
2. Kerkhof, F.P.J.M.; Moulijn, J.A. Quantitative Analysis of XPS Intensities for Supported Catalysts. J. Phys. Chem. **1979**, 83, 1612–1619, doi:10.1021/j100475a011.
3. Shinotsuka, H.; Tanuma, S.; Powell, C.J.; Penn, D.R. Calculations of Electron Inelastic Mean Free Paths. X. Data for 41 Elemental Solids over the 50 eV to 200 keV Range with the Relativistic Full Penn Algorithm. Surf. Interface Anal. **2015**, 47, 871–888, doi:10.1002/sia.5789.
4. Scofield, J.H. Hartree-Slater Subshell Photoionization Cross-Sections at 1254 and 1487 eV. J. Electron Spectrosc. Relat. Phenom. **1976**, 8, 129–137, doi:10.1016/0368-2048(76)80015-1.
5. Tzevelekidis, P.; Theodosiou, M.; Papadopoulou, A.; Sakellis, E.; Boukos, N.; Bikogiannakis, A.K.; Kyriakou, G.; Efthimiadou, E.K.; Mitsopoulou, C.A. Visible-Light-Activated Antibacterial and Antipollutant Properties of Biocompatible Cu-Doped and Ag-Decorated TiO2 Nanoparticles. Heliyon **2024**, 10, e35634, doi:10.1016/j.heliyon.2024.e35634.
6. Larina, T.V.; Dovlitova, L.S.; Kaichev, V.V.; Malakhov, V.V.; Glazneva, T.S.; Paukshtis, E.A.; Bal'zhinimaev, B.S. Influence of the Surface Layer of Hydrated Silicon on the Stabilization of Co2+ Cations in Zr–Si Fiberglass Materials According to XPS, UV-Vis DRS, and Differential Dissolution Phase Analysis. RSC Adv. **2015**, 5, 79898–79905, doi:10.1039/C5RA12551K.
7. Morgan, D.J. Photoelectron Spectroscopy of Ceria: Reduction, Quantification and the Myth of the Vacancy Peak in XPS Analysis. Surf. Interface Anal. **2023**, 55, 845–850, doi:10.1002/sia.7254.
8. Bumajdad, A.; Nazeer, A.A.; Al Sagheer, F.; Nahar, S.; Zaki, M.I. Controlled Synthesis of ZrO2 Nanoparticles with Tailored Size, Morphology and Crystal Phases via Organic/Inorganic Hybrid Films. Sci. Rep. **2018**, 8, 3695, doi:10.1038/s41598-018-22088-0.
9. Liu, M.; Liu, W.; Liu, X.; Ouyang, Y.; Hou, H.; Lei, M.; Wei, Z. Yttrium Oxide as a Q-Switcher for the near-Infrared Erbium-Doped Fiber Laser. Nanophotonics **2020**, 9, 2887–2894, doi:10.1515/nanoph-2019-0563.
10. Park, I.-S.; Jung, Y.C.; Seong, S.; Ahn, J.; Kang, J.; Noh, W.; Lansalot-Matras, C. Atomic Layer Deposition of Y2O3 Films Using Heteroleptic Liquid (iPrCp)2Y(iPr-Amd) Precursor. J. Mater. Chem. C **2014**, 2, 9240–9247, doi:10.1039/C4TC01405G.
11. Tsatsos, S.; Vakros, J.; Ladas, S.; Verykios, X.E.; Kyriakou, G. The Interplay between Acid-Base Properties and Fermi Level Pinning of a Nano Dispersed Tungsten Oxide - Titania Catalytic System. J. Colloid Interface Sci. **2022**, 614, 666–676, doi:10.1016/j.jcis.2022.01.112.
12. Bêche, E.; Charvin, P.; Perarnau, D.; Abanades, S.; Flamant, G. Ce 3d XPS Investigation of Cerium Oxides and Mixed Cerium Oxide (CeTiO). Surf. Interface Anal. **2008**, 40, 264–267, doi:10.1002/sia.2686.
13. Gross, Th.; Ramm, M.; Sonntag, H.; Unger, W.; Weijers, H.M.; Adem, E.H. An XPS Analysis of Different SiO2 Modifications Employing a C 1s as Well as an Au 4f7/2 Static Charge Reference. Surf. Interface Anal. **1992**, 18, 59–64, doi:10.1002/sia.740180110.
